# Onychomycosis Caused by Aspergillus niger in an Immunocompetent Young Female: A Case Report of a Rare Presentation

**DOI:** 10.7759/cureus.87003

**Published:** 2025-06-29

**Authors:** Abdulrahman Saleh Aldairi, Faris Alsaedi, Homaid Alotaibi

**Affiliations:** 1 Department of Dermatology, King Faisal Hospital, Ministry of Health, Makkah, SAU

**Keywords:** aspergillus niger, dermatophytes, fingernail abnormailities, non-dermatophytes, onychomycosis, rare skin disease

## Abstract

Onychomycosis is a fungal infection of the nail that may result from dermatophytes, non-dermatophyte molds, or yeasts. Among non-dermatophyte molds, Aspergillus niger is a recognized species with the potential to cause nail infections. While such infections were traditionally reported in immunocompromised individuals, they have also been increasingly observed in immunocompetent hosts. This condition carries clinical importance due to its potential for recurrence and resistance to treatment. Herein, we present a case of a 25-year-old immunocompetent female with a single fingernail onychomycosis caused by A. niger.

## Introduction

Onychomycosis is a common fungal infection of the nail, most frequently caused by dermatophytes, followed by non-dermatophyte molds (NDMs) and yeasts [1-3]. While dermatophytes such as Trichophyton rubrum remain the predominant cause, increasing reports of NDMs, such as Scopulariopsis brevicaulis, Fusarium, Acremonium, and various Aspergillus species, are reshaping our understanding of fungal nail infections [3,4]. Historically, NDMs were regarded as contaminants or opportunistic organisms with limited clinical significance. However, emerging data suggest a growing role for NDMs, including Aspergillus niger, in causing true nail infections, even in immunocompetent individuals [5]. Aspergillus species are estimated to account for 2.6% to 6.1% of onychomycosis cases globally [3], with Aspergillus niger being a rare isolate and often underreported [1,2,6,7]. The diagnosis of NDM onychomycosis remains a clinical challenge due to overlapping features with dermatophyte infections and the need for repeated, confirmed culture-based identification. Treatment can also be problematic, given the potential for reduced responsiveness to conventional antifungals and the absence of standardized regimens. In this report, we present a case of A. niger-induced fingernail onychomycosis in a 25-year-old immunocompetent woman. We aim to highlight the diagnostic considerations and therapeutic strategies relevant to such rare presentations, based on both available literature and clinical experience.

## Case presentation

**Figure 1:**
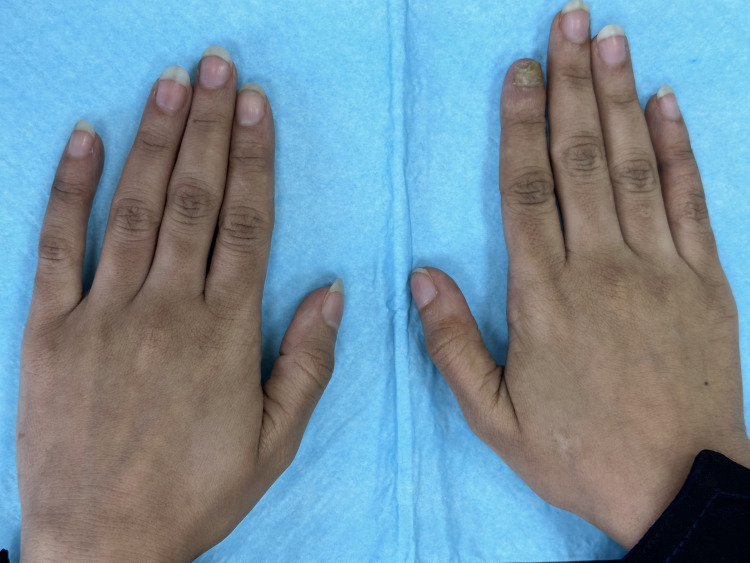
Clinical evidence of onychomycosis affecting the right index fingernail, with all other fingernails appearing normal.

A 25-year-old female patient presented to our Dermatology clinic with an eight-month history of nail deformity with rough surface areas on the right index finger (Figure 1).

**Figure 2:**
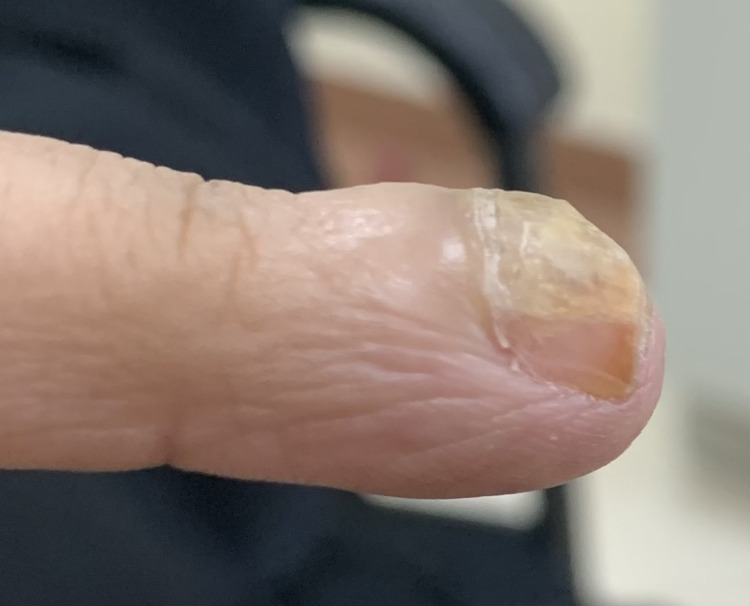
The right index fingernail exhibited dark discoloration at the lateral distal portion of the nail plate, along with overall dystrophic changes.

Apart from that, the patient was in good health, with a history of trauma or nail abnormalities before the onset of the lesion. Her past medical history and family history did not reveal any noteworthy conditions. Upon physical examination, the right index finger had a dark discoloration of the lateral proximal part of the nail plate with marked hyperkeratosis all over the nail (Figure 2).​​​​​​​

The remaining nails of both hands appeared normal, and there were no abnormalities observed in the toenails, the skin of the foot, or the interdigital spaces of the foot. The patient denied any hobbies or occupational exposure involving soil, such as gardening or agricultural work. She is employed in an office-based setting with no history of environmental contact that could predispose her to fungal infection. Laboratory studies, including a complete blood count with differential, random blood glucose, and HbA1c levels, were all within normal limits. Nail sampling technique has been used for direct microscopic examination with 10% potassium hydroxide (KOH) and showed branched non-septated hyphae invade the inside of nail keratin tissue, with no spores or budding cells can be seen hidden under the distorted keratin tissue. This finding is highly related to onychomycosis infection. Treatment was started with amorolfine nail lacquer 5% (applied twice per week) for three months. On the next visit, there was no improvement. Subsequently, we added an oral terbinafine (250 mg), once daily for six weeks, in addition to terbinafine 1% cream (applied twice daily). During the previous treatment course, a fungal culture was performed using Sabouraud dextrose agar (SDA). After two weeks of incubation at room temperature (23-25 °C), there was positive growth of a filamentous, cottony fungus characterized by black, filamentous hyphae resembling small plant-like structures. Additionally, macroscopic observation of the fungal growth on the SDA plate (front view) showed that the colony was initially white to yellow during the first week, later turning black after a few days, with the development of hyphae and conidial spores. On the other hand, the back of the plate and the edges of the colonies appear pale yellow, producing radial fissures. Based on the previous information provided, the isolated black mold was suggestive of A. niger. It's worth mentioning that the previous treatment course did not successfully address the nail abnormalities, and we changed the treatment plan to pulse therapy with oral itraconazole (200 mg twice daily), with instructions for the patient to take it for one week each month over a total of three months. This led to a full resolution of the nail abnormalities, and the patient was subsequently followed for an additional three months. During this period, no recurrence or residual symptoms were observed, confirming sustained clinical and mycological cure.​​​​​​​

## Discussion

Onychomycosis stands as one of the most widespread nail diseases, comprising about 50% of all nail onychopathies [8]. The percentage of cases resulting from non-dermatophytic molds accounts for approximately 1.45% to 17.6% of all cases, with Aspergillus, Scopulariopsis, Fusarium, and Acremonium among the potential causative agents [5]. Almost 900 species were identified in the genus Aspergillus, which are found commonly in the soil and decaying vegetation across the globe. Additionally, they can also be found in various forms of organic debris. Based on the sites mentioned, toenails are 25 times more susceptible to infection compared to fingernails, and this rarity makes it extremely uncommon [9]. Out of these 900 species, A. fumigatus, A. flavus, A. niger, A. terreus, A. glaucus, A. chevalieri, A. ustus, and A. nidulans are recognized as causing infections in the human body [10]. Multiple factors predispose to an increase in disease incidence, including diabetes mellitus, nail trauma, poor nail hygiene, and any disease that results in reduced peripheral circulation. Onychomycosis itself is not considered life-threatening, but it may lead to significant clinical consequences such as chronicity, secondary bacterial infections, and therapeutic difficulties. In addition to serving as a reservoir of infection, if left untreated, it can potentially culminate in permanent nail deformity [11]. Generally, Aspergillus onychomycosis is a distal subungual infection. It typically begins underneath the nail, often near the fingertip, where spores may have become trapped, or laterally, where the nail forms creases in the skin. Unlike some other fungal causes of nail infection, the fungus will not spread to the surrounding skin in this case. Aspergillus species found in natural environments often produce vibrant pigments. Consequently, an Aspergillus nail infection can manifest as greenish, black, brown, or other discolorations [12]. We believe that color discoloration in the fingernail or toenail can be an excellent clue to raise suspicion of Aspergillus, but it is not a diagnostic tool. In the literature, we found a case reported by Tamer et al. [6] demonstrated that there was no toenail pigmentation in their patient. Accordingly, A dermatologist cannot diagnose Aspergillus onychomycosis solely by visual examination of the affected nail [12]. Hence, a fungal culture on artificial media must be performed for a definitive diagnosis of Aspergillus onychomycosis. It is important to note that the standardization of the treatment of non-dermatophytic onychomycosis is not well established. Moreover, treatment is difficult due to the rigidity of the nail plate and the anatomical location of the infection between the nail bed and plate. However, several research studies indicate varying treatment approaches yielding diverse outcomes, with effective treatment responses observed when treating Aspergillus spp., especially when compared to other non-dermatophytic molds [13]. For example, Tosti et al. [13] used a combination of topical and systemic therapy in cases of onychomycosis caused by Aspergillus spp., resulting in complete cure in five out of seven patients. While Gianni and Romano [14] conducted a research study involving 34 patients diagnosed with Aspergillus onychomycosis, these patients were subjected to treatment with oral terbinafine at a daily dose of 500 mg, following a regimen consisting of one week of administration followed by a three-week suspension, with the entire treatment pulse regimen spanning 3 months. Consequently, an 88% cure rate was attained. Tosti and Piraccini [7] documented two cases in which oral terbinafine was administered at a daily dose of 250 mg for three months, resulting in a full recovery. The length of the treatment period varies depending on the affected anatomical location, with affected fingernails typically requiring a three-month therapy, whereas toenails typically need a minimum of six months of treatment [15]. In our case, we initially administered oral terbinafine in addition to topical treatments, but there was no response. Thus, after reviewing the literature, we identified some research studies that suggest using itraconazole as a pulse therapy, as it appears to yield better results compared to terbinafine [15,16]. However, with this treatment strategy, our patient achieved complete recovery.

## Conclusions

This case highlights the importance of considering non-dermatophyte molds, particularly A. niger, as potential causative agents of onychomycosis even in immunocompetent individuals. Accurate identification through appropriate fungal culture and microscopic examination is essential for guiding effective management. Clinicians should maintain a high index of suspicion for atypical organisms, especially in cases unresponsive to standard antifungal treatments. Early diagnosis and targeted therapy can lead to successful outcomes and reduce the risk of chronic infection or recurrence. This report emphasizes the need for increased awareness of emerging fungal pathogens in nail infections and the importance of individualized treatment planning.
